# ω‐3PUFAs Inhibit Gallbladder Cancer Through Enhancing STK31 Methylation

**DOI:** 10.1002/mnfr.70522

**Published:** 2026-06-11

**Authors:** Shuang‐Xing Li, Wei‐Yu Zhu, Hai‐Ying Peng, Bin Zhang, Zhu‐Ying Lin, Song‐Lin Yang, Yi‐Yi Zhao, Hui Gao, Shan He, Yuan Liu, Xiao‐Dan Wang, Yang‐Fan Guo, Wen‐Ju Wang, Zong‐Liu Hou, Ming‐Yao Meng, Li‐Wei Liao, Lin Li

**Affiliations:** ^1^ Central Laboratory of Yan'an Hospital Affiliated to Kunming Medical University Kunming Yunnan Province China; ^2^ Key Laboratory of Tumor Immunological Prevention and Treatment of Yunnan Province Kunming Yunnan Province China; ^3^ Kunming Medical University Kunming Yunnan Province China

**Keywords:** ω‐3PUFAs, DNA methylation, gallbladder cancer, STK31, GSK3β

## Abstract

Gallbladder cancer (GBC) is an aggressive malignancy with limited treatment options. ω‐3PUFAs have known anticancer activity, but their role in GBC remains unclear. The anti‐GBC effects of ω‐3PUFAs (DHA/EPA) were evaluated using in vitro assays and mouse xenograft models. Epigenetic changes were analyzed by RRBS, MSP, and MethyLight PCR. Downstream mechanisms were explored via proteomics, Co‐IP, and Western blot. This study demonstrates that ω‐3PUFAs suppress GBC progression by inducing promoter hypermethylation of STK31, which is an oncogene found in GBC by our previous study. MethyLight PCR revealed that ω‐3PUFAs treatment significantly increased STK31 promoter methylation in GBC‐SD cells from 34.1% ± 20.91% to 89.1% ± 13.11%. Moreover, STK31(DNMT1)/GSK3β pathway and COL4A1 (ITGB1) were particularly associated with ω‐3PUFAs' inhibitory mechanisms to GBC. In vivo experimental results demonstrated that ω‐3PUFAs significantly inhibited the growth of gallbladder cancer xenografts, with tumor growth inhibition rates of 38.6% for GBC‐SD and 50.2% for NOZ at day 33, and effectively suppressed tumor cell metastasis in an intraperitoneal mouse model. This study provides first evidence that ω‐3PUFAs target STK31 via epigenetic reprogramming, opening new avenues for dietary intervention and targeted therapy of GBC.

## Graphical abstract

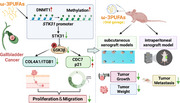

**Abbreviations**
5‐Aza: 5‐azacytidine
ALA: alpha‐linolenic acid
ANOVA: analysis of variance
BCA: bicinchoninic acid
CCK‐8: cell counting kit‐8
CDC7: cell division cycle 7
ChIP: chromatin immunoprecipitation
Co‐IP: co‐immunoprecipitation
COL4A1: collagen Type IV Alpha 1 Chain
COL4A2: collagen Type IV Alpha 2 Chain
DEPs: differential expression proteins
DHA: docosahexaenoic acid
DNMT1: DNA methyltransferase 1
DNMT3B: DNA methyltransferase 3B
ECL: enhanced chemiluminescence
EPA: eicosapentaenoic acid
GBC: gallbladder cancer
GSEA: gene set enrichment analysis
GSK3β: glycogen synthase kinase 3 beta
IF: immunofluorescence
IHC: immunohistochemistry
ITGB1: integrin subunit beta 1
ITGB2: integrin subunit beta 2
IVIS: in vivo imaging system
KEGG: kyoto encyclopedia of genes and genomes
MSP: methylation‐specific PCR
NOZ‐luc: NOZ‐luciferase
p21: cyclin‐dependent kinase inhibitor 1a
PCA: principal component analysis
RRBS: reduced representation bisulfite sequencing
RT‐qPCR: quantitative real‐time PCR
STK31: serine/threonine kinase 31
ω‐3PUFAs: omega‐3 polyunsaturated fatty acids

## 1 Introduction

Gallbladder cancer (GBC) is the most common malignant tumor of the biliary system, with a 5‐year survival rate of 5% and a lack of effective treatments for advanced patients [1]. Surgery remains the sole curative option for GBC, but it is feasible mainly in early‐stage disease and carries a high risk of recurrence. In advanced cases, systemic chemotherapy constitutes the primary treatment modality; however, its therapeutic benefit is limited and often accompanied by substantial toxicity [2]. Therefore, research efforts aimed at preventing and treating GBC are of great importance.

**FIGURE 1:**
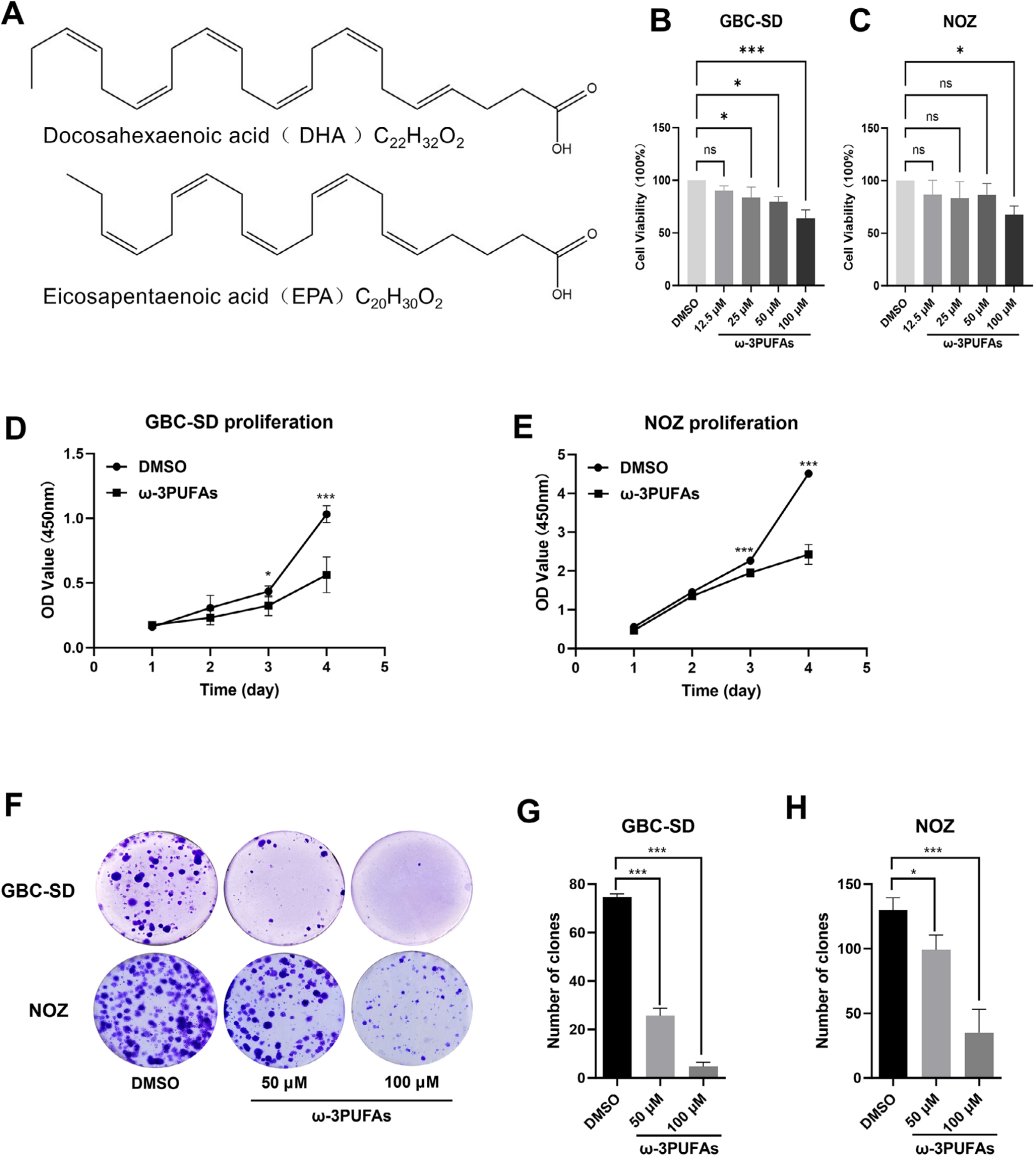
(A) The structures of DHA and EPA. (B–H) The inhibitory effects of ω‐3PUFAs (DHA and EPA) on the viability and clone formation of GBC‐SD and NOZ cells. Gallbladder cancer cells were incubated with 12.5, 25, 50, or 100 µM ω‐3PUFAs for 24 h, and with 100 µM ω‐3PUFAs for 24, 48, 72, or 96 h in another set of experiments. Cell viability was detected by CCK8 assay with three independent biological replicates. (F) The representative photos of clone formation assay. (G and H) Quantification of colony numbers from four independent biological replicates. All data were presented as mean ± SD. *p < 0.05, ***p < 0.001 comparing ω‐3PUFAs treated cells with vehicle control cells by Student's *t*‐test or one‐way ANOVA followed by post hoc Dunnett's test.

Among the bioactive components of fish oil, omega‐3 polyunsaturated fatty acids (ω‐3PUFAs) have attracted increasing scientific attention. Current evidence suggests that, in contrast to ω‐6PUFAs, which have been linked to a potential elevation in cancer risk, ω‐3PUFAs may exert protective effects and hold promise for cancer prevention and therapy [3]. ω‐3PUFAs, a subclass of polyunsaturated fatty acids within the linolenic acid family, primarily include eicosapentaenoic acid (EPA), docosahexaenoic acid (DHA) (Figure 1A), and alpha‐linolenic acid (ALA). The FDA has approved ω‐3PUFAs as a prescription drug for the treatment of hypertriglyceridemia, and multiple clinical trial results have also demonstrated their efficacy [4, 5]. Previous studies revealed that ω‐3PUFAs exhibited potential protective effects against gallstones, cholecystitis, and obesity by reducing cholesterol saturation [6, 7] and demonstrated the dissolution of cholesterol gallstones in murine model [8]. Furthermore, the issues mentioned above are also risk factors for GBC.

An increasing body of evidence indicates that ω‐3PUFAs confer protective/beneficial effects against a variety of diseases, potentially through epigenetic mechanisms—most notably DNA methylation—that play a pivotal role in regulating gene expression [9, 10]. Our previous study found that serine/threonine kinase 31 (STK31), a novel cancer‐testis antigen (CTA), was closely associated with the development and progression of GBC. Suppression of STK31 expression has been shown to significantly inhibit GBC growth and metastasis. Furthermore, *STK31* harbors 37 CpG sites within its promoter/exon 1 region, and its expression was regulated by promoter methylation [11, 12]. Demethylation‐induced activation of *STK31* has been linked to cervical, gastric, colorectal, and esophageal cancers [11, 12, 13], but its relevance in GBC remains unclear. Therefore, we hypothesize that ω‐3PUFAs may exert their inhibitory effect on GBC by modulating the methylation level of *STK31*. To validate this scientific hypothesis, we employed reduced representation bisulfite sequencing (RRBS) to analyze the methylation profiles of GBC cells following ω‐3PUFAs treatment. Meanwhile, cell‐based in vitro models and multiple tumor xenograft mouse models were applied to verify the inhibitory effect of ω‐3PUFAs on GBC. Additionally, proteomics was utilized to identify downstream molecules through which ω‐3PUFAs act via STK31. Finally, the underlying mechanism of action was validated.

## 2 Materials and Methods

### 2.1 Materials

Human GBC cell lines GBC‐SD and NOZ were purchased from the Cell Bank of Type Culture Collection of Chinese Academy of Sciences. Primary antibody against STK31 for western blot was purchased from Biobyt, Cambridge, UK. Antibodies against integrin subunit beta 2 (ITGB2), glycogen synthase kinase 3 beta (GSK3β), p‐GSK3β(Ser9), collagen type IV alpha 1 chain (COL4A1), cell division cycle (CDC) 7 were obtained from Cell Signaling Technology, MA, USA. Antibodies against integrin subunit beta 1 (ITGB1), DNA methyltransferase 1 (DNMT1), β‐actin, DNA methyltransferase 3B (DNMT3B), and collagen type IV alpha 2 Chain (COL4A2) were purchased from Proteintech, Wuhan, China.

### 2.2 Dosage Information/Dosage Regimen

DHA, EPA (Figure 1A) and corn oil were purchased from MedChem Express, Shanghai, China. DHA and EPA were dissolved in DMSO (Solarbio, Beijing, China) at a 1:1 molar ratio (ω‐3PUFAs mixture) to prepare a 20 mM stock solution, which were stored at −80°C for subsequent use.

### 2.3 Cell Culture

GBC‐SD and NOZ cells were cultured in RPMI 1640 and Dulbecco's Modified Eagle Medium (DMEM)/F12 (Gibco, Thermo Fisher Scientific (SuZhou) Instruments, USA), respectively, and supplemented with 10% *v/v* fetal bovine serum (FBS, BI, Israel), 100 U/ml penicillin, and 10 U/ml streptomycin (Vivacell, Shanghai, China), and maintained at 37°C in a humidified incubator with 5% CO_2_.

### 2.4 Cell Counting Kit (CCK)‐8 and Colony Formation Assays

GBC‐SD and NOZ cells were seeded in 96‐well plates at a density of 5 × 10^3^ cells per well. To evaluate the dose‐dependent effects of ω‐3PUFAs, cells were treated with vehicle control (DMSO), 12.5, 25, 50, or 100 µM ω‐3PUFAs for 24 h. To evaluate the time‐dependent effects, cells were treated with 100 µM ω‐3PUFAs for 24, 48, 72, or 96 h. To mitigate the potential impact of the solvent on cell growth, the final concentration of DMSO in all culture wells was maintained below 0.05%. After the treatment, the absorbance of the cells was detected, respectively at 450 nm using the CCK‐8 kit (Proteintech, Wuhan, China). 10 µL of CCK‐8 reagent was added to each well, and the cells were incubated in the dark at 37°C for 2 h. The actual absorbance value was detected by a microplate spectrophotometer (Varioskan Lux, Thermo Scientific, USA).

Cells were plated in 6‐well plates at the following densities: NOZ, 5 × 10^2^ cells/well; GBC‐SD, 1 × 10^3^ cells/well. Subsequently, 50 and 100 µM ω‐3PUFAs were added into the cells for 10 or 12 days, respectively. After fixation with methanol, the colonies were stained with 1% crystal violet, air‐dried, subsequently documented by photography, and counted.

### 2.5 Transwell Cell Migration Assay

The cell migration ability of GBC cells was evaluated by the Boyden chamber assay. Briefly, GBC‐SD and NOZ cells were seeded into each transwell filter chamber with 8 µm pore size (Corning, USA) at a density of 2 × 10^4^ cells per well. Concurrently, 50 µM and 100 µM ω‐3PUFAs were prepared in 100 µL of low‐serum medium (containing 0.5% *v/v* FBS) and added to the upper chambers for a co‐incubation period of 16–18 h. Complete medium (10% *v/v* FBS) was placed in the lower chamber to function as the chemoattractant. Cells that had migrated to the lower chamber of the membrane were fixed with methanol and stained with crystal violet. The cells were photographed under a microscope (Primo Vert, Carl Zeiss Microscopy GmbH, Germany), and counted using ImageJ software.

### 2.6 Cell Cycle Analysis by Flow Cytometry

GBC‐SD and NOZ cells were cultured in 10‐cm cell culture dish at a cell count of 1 × 10^6^ cells per dish. After being treated with DMSO, 100 µM or 125 µM ω‐3PUFAs for 48 h, the cells were fixed with 75% ethanol and stained with propidium iodide (PI)/RNase A. Red fluorescence was detected at the excitation wavelength of 488 nm using a flow cytometer (FACSCanto II, BD, USA), and the light scattering was also detected simultaneously. The cellular DNA content was analyzed using the Modfit analysis software.

### 2.7 Reduced Representation Bisulfite Sequencing (RRBS)

Previous studies suggested the potential of ω‐3PUFAs to modulate gene methylation levels [9, 10]. Hence, we utilized RRBS to assess the effects of ω‐3PUFAs on the DNA methylome of GBC cells. GBC‐SD cells (5 × 10^6^ cells) were treated with DMSO or 100 µM ω‐3PUFAs for 24 h, and genomic DNA of cells was extracted. Following DNA fragmentation, the library was prepared through end repair, 3′ adenylation, and adapter ligation. Subsequently, bisulfite conversion was performed using the EZ DNA Methylation‐Gold Kit (Zymo Research, California, USA). Prior to library construction, a set proportion of human mitochondrial DNA was added as a negative control to calculate the bisulfite conversion rate. Following this, the libraries underwent size selection (200–500 bp insert range) and PCR amplification. Sequencing was performed on the HiSeq 4000 platform using a 150‐base pair paired‐end (150 bp PE) strategy. Differential methylation analysis and correlation assessments were performed using the R package MethylKit. To examine specific genomic regions, the data were visualized and analyzed with IGV software, specifically targeting the *STK31* promoter at locus 7p15.3 on the autosomal chromosome.

### 2.8 Methylation‐Specific PCR (MSP) and MethyLight PCR

To further clarify the effect of ω‐3PUFAs on the methylation of the *STK31* promoter, we performed MSP and MethyLight PCR for validation. After GBC‐SD and NOZ cells (1 × 10^6^ cells) were treated with DMSO and ω‐3PUFAs (100 or 125 µM) for 24 h, total DNA was extracted by TIANamp Genomic DNA Kit (TIANGEN, Beijing, China). Then bisulfite conversion of DNA was carried out by EpiArt Ultrafast DNA Methylation Bisulfite Kit (Vazyme, Nanjing, China) according to the manufacturer's instructions. The bisulfite‐treated DNA as a template with methylation‐specific and/or unmethylation‐specific primers were performed MSP. After amplification with methylation‐specific primers, the methylation status was determined by 1% agarose gel electrophoresis. Methylation‐specific primers and unmethylation‐specific primers were as follows: methylated *STK31*: F: 5ʹ TTGTTACGTGATTTTCGTTAATATC‐3ʹ and R: 5ʹ TAAACCCACATACTAAACTTTCGAC‐3ʹ; unmethylated *STK31*: F: 5'‐GTTATGTGATTTTTGTTAATATT‐3' and R: 5ʹ TAAACCCACATACTAAACTTTCAAC‐3ʹ.

For MethyLight PCR, 1 × 10^6^ GBC‐SD cells were incubated with 100 or 125 µM ω‐3PUFAs for 24 h. The processes of DNA extraction and bisulfite conversion were the same as those described for the MSP. The methylation levels of *STK31* were quantified using TaqMan probes (Table S1) and 2 × T5 Fast qPCR Mix (Probe) from Tsingke Biotechnology Co., Ltd., Beijing, China.

### 2.9 Quantitative Real‐Time PCR (RT‐qPCR)

RT‐qPCR was employed to measure the mRNA expression levels of the *STK31* and *DNMT1* genes in response to ω‐3PUFAs treatment. GBC‐SD and NOZ cells (1 × 10^6^ cells/ dish) were treated with 100 or 125 µM ω‐3PUFAs for 24 h, and the total RNA was extracted by Eastep Super Total RNA Extraction Kit and reverse‐transcribed into cDNA using GoScript Reverse Transcription Mix, Oligo(dT) (Promega Biotech, Beijing, China). RT‐qPCR was performed using SsoFast EvaGreen Supermix according to the manufacturer's instructions (Bio‐Rad, CA, USA). The relative gene expression was calculated using the 2−ΔΔ*C*_t_ method. The primers used were as follows: *GAPDH*: F:5′‐GGAGCGAGATCCCTCCAAAAT‐3′, R:5'‐GGCTGTTGTCATACTTCTCATGG‐3′; *STK31*: F:5′‐TGAACTCTGGTGGTGGTCTCCTTAC‐3′, R:5′‐CTTGGCTTCTGTGTCAACATCC‐3′; *DNMT1*: F:5′‐CCGAGTTGGTGATGGTGTGT‐3ʹ, R: 5ʹ‐CCGTTGCTCTTCTTGGGACA‐3ʹ.

To further validate that ω‐3PUFAs suppress *STK31* expression by promoting its methylation, we treated the GBC‐SD cells with ω‐3PUFAs in the presence or absence of the methylation inhibitor 5‐azacytidine (5‐Aza) and measured *STK31* mRNA levels using RT‐qPCR. Briefly, GBC‐SD cells were seeded in 10‐cm culture dishes at a density of 1 × 10^6^ cells per dish. After cell adhesion, the cultures were treated with DMSO, 5 µM 5‐Aza, 100 µM ω‐3PUFAs, or a combination of 5 µM 5‐Aza and 100 µM ω‐3PUFAs for 24 h. Subsequently, the cells were harvested for RNA extraction and RT‐qPCR.

### 2.10 Western Blot

GBC‐SD and NOZ cells were seeded at a density of 1 × 10^6^ cells per dish overnight, and then treated with 100 or 125 µM ω‐3PUFAs for 24 h. Proteins were extracted using RIPA buffer containing protease and phosphatase inhibitors (Roche Molecular Biochemicals, Switzerland). Protein concentrations were measured using a bicinchoninic acid (BCA) assay kit from Beyotime (Shanghai, China). Then protein aliquots of 30 µg were followed by SDS‐PAGE electrophoresis (80 V, 100 min) and membrane transfer (200 mA, 100 min). Following blockage with 5% (*w*/*v*) non‐fat dry milk in TBS for 1 h, the PVDF membrane (ISEQ00010, Millipore, Molbach, Germany) was subsequently subjected to an overnight exposure to primary antibodies for STK31, DNMT1, GSK3β, p‐GSK3β (Ser9), COL4A1, COL4A2, ITGB1, ITGB2, cyclin‐dependent kinase inhibitor 1a (p21), DNMT3B, and CDC7 (1:1000) at 4°C. After incubation with an appropriate secondary antibody, enhanced chemiluminescence (ECL) (PK10003, Proteintech, Wuhan, China) detection was performed, and ImageJ was used for quantification. To confirm equal protein loading, the membranes were also incubated with antibody against β‐actin (1:5000).

### 2.11 Chromatin Immunoprecipitation (ChIP)

We employed ChIP‐qPCR to investigate whether the enrichment of DNMT1 at the *STK31* promoter region was affected by ω‐3PUFAs treatment. GBC‐SD cells were seeded at a density of 2 × 10^6^ cells per dish and treated with DMSO, 100 or 125 µM ω‐3PUFAs for 24 h. The cells were then cross‐linked with formaldehyde, and chromatin was sonicated to fragment it into 200–1000 bp. ChIP assay was performed using the Pierce Magnetic ChIP Kit (ThermoFisher Scientific, MA, USA) according to the manufacturer's instructions. Immunoprecipitation was performed using an antibody against DNMT1 (5032, Cell Signaling Technology, MA, USA). Normal rabbit IgG (30000‐0‐AP, Proteintech, Wuhan, China) and input DNA were included as controls. The enrichment of the *STK31* promoter was quantified using SYBR‐Green Real‐Time qPCR. *STK31* promoter primer sequence was as follows: *STK31* promoter forward (F), 5′‐ATGCTGACAAATTGCTTCTACGA‐3′ and reverse (R), 5′‐GGATTCTCTGTTCTGTTACACTGA‐3′.

### 2.12 Proteomics and Bioinformatics Analysis

The proteomics was employed to further elucidate the downstream molecular mechanisms by which ω‐3PUFAs inhibit the proliferation and migration of GBC through STK31. GBC‐SD cells (1 × 10^6^ cells/dish) were exposed to ω‐3PUFAs for 24 h, then total proteins from treated‐cells were extracted by the lysis buffer (1% SDC and 1% protease inhibitor) and subjected to enzymatic digestion. The protein concentration was determined using BCA assay. Peptide segments were separated using the NanoElute ultra‐high‐performance liquid chromatography system. After separation, the peptide segments were injected into a Capillary ion source for ionization and then subjected to data acquisition using the timsTOF Pro 2 mass spectrometer. The DIA data were processed using DIA‐NN search engine (v.1.8). Tandem mass spectra were searched against the Homo_sapiens_9606_SP_20231220.fasta (20429 entries) concatenated with reverse decoy database. Differential expression proteins (DEPs) were screened using |FC| ≥ 1.5 and *p* < 0.05 as the criteria. Principal component analysis (PCA) was performed using the prcomp function from the stats package in R, and the results were visualized using the ggplot2 and ggpubr packages. Heatmap visualization and clustering were conducted with the ComplexHeatmap package in R. For row clustering, Euclidean distance was used as the distance measure (clustering_distance_rows = “euclidean”), and complete linkage was used as the agglomeration method (clustering_method_rows = “complete”). Protein pathways were annotated using the Kyoto Encyclopedia of Genes and Genomes (KEGG) database, with protein identification achieved via BLASTP (*E*‐value ≤1 × 10^−4^), assigning the top‐hit annotation to each query sequence. In addition, we performed bioinformatics analysis on a previously generated dataset from STK31‐knockdown GBC‐SD cells, which was obtained using 4D label‐free quantitative proteomics (based on dda‐PASEF mode), whereas the current study used 4D Fast DIA (based on dia‐PASEF mode). Despite the different acquisition modes, both datasets were generated using the same cell line, same culture conditions, same timsTOF Pro 2 platform, and processed with identical DEP screening criteria (|FC| ≥ 1.5, *p* < 0.05), ensuring comparability. The Venn diagram was generated using an online platform for data analysis and visualization (https://www.bioinformatics.com.cn).

### 2.13 Immunofluorescence (IF)

Cells were fixed with 4% paraformaldehyde for 15 min and permeabilized with 0.5% Triton X‐100 (prepared in PBS) at room temperature for 20 min. Cells were then blocked with 5% horse serum (SL042, Solarbio, Beijing, China) and incubated with anti‐GSK3β antibody (67329‐1‐Ig, Proteintech, Wuhan, China; 1:250) and anti‐STK31 antibody (PA5‐31551, Invitrogen, Carlsbad, CA, USA; 1:250) overnight at 4°C. Next, cells were incubated with Alexa Fluor 488 goat anti‐rabbit IgG (H+L) cross‐adsorbed secondary antibody (A110008, Invitrogen, USA, 1:200) and Alexa Fluor 594 conjugated goat anti‐mouse IgG (H+L) (SA00006‐3, Proteintech, China; 1:200). Finally, cell fluorescence was detected and analyzed using a ZEISS LSM900 confocal microscope. Colocalization analysis was performed using ZEISS ZEN 3.8 software.

### 2.14 Phos‐Tag SDS‐PAGE and Transfer

Since STK31 is a kinase, we employed Phos‐tag SDS‐PAGE to determine whether it regulates GSK3β through phosphorylation. Protein STK31 was Flag‐tagged and GSK3β was HA‐tagged through transfecting plasmids GV492 (pcDNA3.1 (STK31)) and GV513 (pcDNA3.1 GSK3β) (Shanghai Genechem) into GBC‐SD cells respectively. Then the cells were lysed and the protein samples were collected with the same procedures as western blot. The protein samples were separated using phosphorylation‐tag (Phos‐tag, 50 µmol/L) SDS‐PAGE precast gel(12.5%)(195‐17991, Wako, Japan) under a constant current of 30 mA per gel. The gel was washed with 5 mmol/L EDTA for 20 min to chelate divalent manganese ions and then with fresh transfer buffer for 15 min. The proteins were transferred from the Phos‐tag gel to a membrane at 250 mA for 90 min. Following blocking, the membrane was incubated with primary and secondary antibodies (HA tag recombinant monoclonal antibody, 1:1000, 81290‐1‐RR; Multi‐rAb HRP‐goat anti‐rabbit recombinant secondary antibody (H+L), 1:5000, RGAR001; Proteintech, Wuhan, China), and the signal was detected by ECL.

### 2.15 Co‐Immunoprecipitation (Co‐IP)

A potential interaction between STK31 and GSK3β was investigated. STK31 was Flag‐tagged and GSK3β was HA‐tagged. The complex was co‐immunoprecipitated with an anti‐Flag antibody and the presence of the HA‐tagged GSK3β in the precipitate was confirmed by western blot. Briefly, GBC‐SD cells (1 × 10^7^) were lysed with IP cell lysis buffer (P0013B, Beyotime Biotechnology, Shanghai, China) containing protease and phosphatase inhibitor mixture (04693132001, Roche, Penzberg, Germany; PR20015, Proteintech, Wuhan, China). Co‐IP was performed using the Catch and Release v2.0 reversible immunoprecipitation system (17–500 M, Merck Millipore, Darmstadt, Germany) according to the manufacturer's instructions. Rabbit anti‐Flag (20543‐1‐AP, Proteintech, Wuhan, China) and Rabbit IgG (30000‐0‐AP, Proteintech, Wuhan, China) primary antibodies were used for immunoprecipitation overnight at 4°C. The immunoprecipitates from the cells were heated at 100°C for 5 min, separated by 10% SDS‐PAGE, and then transferred to PVDF membranes. The membranes were blocked with 5% BSA in TBST at room temperature for 1 h, followed by incubation with primary antibodies: mouse anti‐Flag (F1804, Sigma, Germany, 1:1000) and mouse anti‐HA (AE008, abclonal, China, 1:1000). Subsequently, the membranes were incubated with multi‐rAb HRP‐Goat anti‐mouse recombinant secondary antibody (H+L) (RGAM001, Proteintech, Wuhan, China, 1:5000) at room temperature for 60 min. The membranes were then visualized using ECL reagent.

### 2.16 In Vivo Xenograft Mouse Studies

The animal studies were conducted in compliance with the guidelines approved by the Animal Experimentation Ethics Committee of the Yan'an Hospital Affiliated to Kunming Medical University (No. AEWC‐2022020). Six‐week‐old male BALB/c nude mice were obtained from SPF Biotechnology Co., Ltd. (Beijing, China) and housed separately bred in pathogen‐free conditions.

#### 2.16.1 The Subcutaneous Xenograft Models

The mice received a daily oral gavage of ω‐3PUFAs (350 mg/kg/day) or corn oil for 7 days, and then followed by subcutaneous inoculation of GBC‐SD cells (5 × 10^6^) or NOZ cells (2 × 10^6^) embedded in Matrigel (Corning, USA). For GBC‐SD xenografts, mice were randomly assigned to two groups: corn oil control (*n* = 13) and ω‐3PUFAs treatment (*n* = 10). For NOZ xenografts, mice were assigned to corn oil control (*n* = 11) and ω‐3PUFAs treatment (*n* = 9). Tumor volume (*V* = 1/2 × length×width^2^) was measured every 3 days. After 34 days of intragastric injection, the mice were euthanized with an overdose of sodium pentobarbital intraperitoneally and sacrificed by cervical dislocation. The tumors were then completely removed, photographed, and weighed. The tumor samples were fixed in 4% paraformaldehyde and further processed for immunohistochemistry (IHC).

#### 2.16.2 An Intraperitoneal Xenograft Model

Given the pronounced anti‐metastatic activity of ω‐3PUFAs observed in vitro (as shown in result section), an intraperitoneal NOZ tumor xenograft mouse model was utilized, as described previously with modification [14], to assess its therapeutic effect. Briefly, NOZ cells were transfected with lentiviral vector carrying the firefly luciferase gene (Genechem Co. Ltd., Shanghai, China). NOZ‐luciferase (NOZ‐luc) cells were established after puromycin selection and validated using ONE‐Glo Luciferase Assay System (E6120, Promega, Madison, USA). The nude mice were also pre‐treated with ω‐3PUFAs (350 mg/kg/day) or corn oil for 7 days, and each mouse was injected intraperitoneally with 1 × 10^6^ NOZ‐luc cells. The control group received corn oil (*n* = 12), and the treatment group received ω‐3PUFAs (*n* = 13). The tumor burden in mice was observed through the in vivo imaging system (IVIS) (AniView100, Bluete, Guangzhou, China) after Beetle Luciferin Potassium Salt (E1605, Promega, Madison, USA) was administered by intraperitoneal injection. In vivo bioluminescence imaging was performed on day 8 post‐cell inoculation and then every 8 days for a total of 3 imaging sessions. Bioluminescent signals from the tumors were captured and results were quantified as the average radiance (p/s/cm^2^/sr).

#### 2.16.3 Immunohistochemistry (IHC)

The fixed tissue specimens were embedded in paraffin and sectioned at 5 µm. After dewaxing and rehydration, the sections were incubated in citrate buffer at 100°C for 10 min for antigen retrieval and incubated with blocking buffer (5% normal goat serum in PBS) for 1 h at room temperature. Then the samples were incubated overnight at 4°C with the primary antibodies, including anti‐STK31 (Invitrogen, Carlsbad, CA, USA), anti‐GSK3β (12456, Cell Signaling Technology, USA) and anti‐Ki‐67 (Abcam, Cambridge, UK). After three washes with PBST (PBS with 0.05% Tween‐20), tissue sections were incubated with biotinylated secondary antibody reagents for 20 min at room temperature. Following three washes with PBS, the sections were incubated with DAB solution (G1212, Servicebio, Wuhan, China) and then counterstained with hematoxylin. Stained sections were visualized and imaged with a light microscope (Carl Zeiss Axioscope 5, Germany).

### 2.17 Statistical Analysis

Tumor weight data are presented as median and interquartile range (IQR). All data in in vitro assays are presented as the mean ± standard deviation (Mean ± SD), and as the means ± S.E.M (Standard error of the mean) for other in vivo studies. Student *t*‐test or the nonparametric Mann–Whitney *U* test was used to compare differences between two groups, and one‐way analysis of variance (ANOVA) was used to compare differences among multiple groups. A Chi–square test was used to compare the distribution differences of various cell cycle levels between two groups or multiple groups. Differences were considered statistically significant when *p* < 0.05. GraphPad Prism 9.0.0 statistical analysis software was used for data analysis. The in vivo fluorescence intensity data were log‐transformed and assessed for normality and homogeneity of variance using the Shapiro–Wilk test.

## 3 Results

### 3.1 ω‐3PUFAs Inhibit the Proliferation of GBC Cells In Vitro

To assess the impact of ω‐3PUFAs on GBC, GBC‐SD and NOZ cells were exposed to 12.5, 25, 50, or 100 µM of ω‐3PUFAs for 24 h. In GBC‐SD cells, ω‐3PUFAs significantly reduced cell viability at 25 and 50 µM, with a more pronounced inhibitory effect observed at 100 µM. In contrast, NOZ cells exhibited a significant reduction in viability only at the highest concentration (100 µM). Cell viability was markedly reduced in a dose‐dependent manner (Figure 1B,C). In addition, compared with the vehicle control group, treatment with 100 µM of ω‐3PUFAs for 24–96 h resulted in a progressive significant reduction in the proliferation of GBC‐SD and NOZ cells, indicating a time‐dependent inhibitory effect (Figure 1D,E). We further performed a colony formation assay to evaluate the effect of ω‐3PUFAs on the long‐term proliferative capacity of GBC cells. GBC‐SD and NOZ cells were treated with 50 or 100 µM ω‐3PUFAs for 10 and 12 days, respectively. ω‐3PUFAs significantly suppressed colony formation in both cell lines (Figure 1F). In GBC‐SD cells, colony formation was reduced by 65.3% ± 2.99% and 93.3% ± 1.71% at 50 and 100 µM, respectively, compared with the DMSO control group. In NOZ cells, the reductions were 23.8% ± 11.5% and 73.1% ± 18.2%, respectively (Figure 1G,H).

### 3.2 ω‐3PUFAs Inhibit the Metastasis of GBC Cells and Induce Cell Cycle Arrest in vitro

**FIGURE 2:**
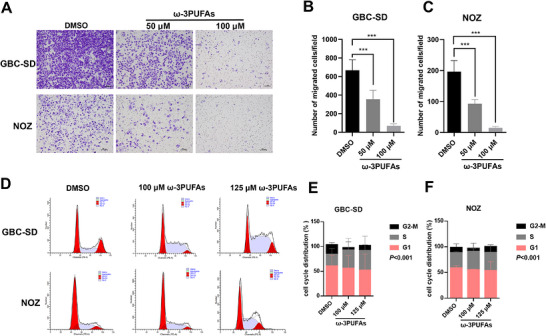
The anti‐migratory effects and cell cycle arrest induced by ω‐3PUFAs on GBC cells (GBC‐SD and NOZ cells). (A) Representative photos showed the stained cells on the lower chamber of the membrane. The above cells migrated to the lower chambers were stained and the numbers were counted. (B and C) Quantification of migration of GBC‐SD and NOZ cells. Differences between treatment groups and control group were determined by one‐way ANOVA followed by post hoc Dunnett's test, ***p < 0.001. (D) Representative images of cell cycle analysis with PI staining followed by flow cytometry. (E and F) The cell cycle distributions of each phase of GBC cells were summarized in bar chart with percentage. All data were presented as mean ± SD from three independent biological replicates. Chi–square test was used to compare the distribution differences of cell cycle between DMSO control group and 100 µM, 125 µM ω‐3PUFAs treatment groups, ***p < 0.001.

After 16–18 h of treatment with ω‐3PUFAs, the number of GBC cells migrating to the lower chamber of the membrane was significantly decreased in the ω‐3PUFAs‐treated groups compared to the vehicle control group, indicating a remarkable reduction in cell migration abilities (Figure 2A). Moreover, cell motilities were significantly reduced by ω‐3PUFAs in a concentration‐dependent manner, with higher concentrations producing a more pronounced inhibitory effect (Figure 2B,C, *p* < 0.001).

Since cell cycle regulation directly affects cell proliferation, we further utilized flow cytometry to analyze the effects of ω‐3PUFAs on the cell cycle of GBC cells. The results demonstrated that after 48 h of treatment with ω‐3PUFAs, the percentage of cells in S phase significantly increased in both GBC‐SD and NOZ cells, while the proportion of cells in G2‐M phase decreased. These findings indicate that the cell cycle was disrupted, leading to S phase arrest (Figure 2D–F).

### 3.3 ω‐3PUFAs Downregulate the Expression of STK31 in GBC Cells by Promoting DNMT1 Expression

**FIGURE 3:**
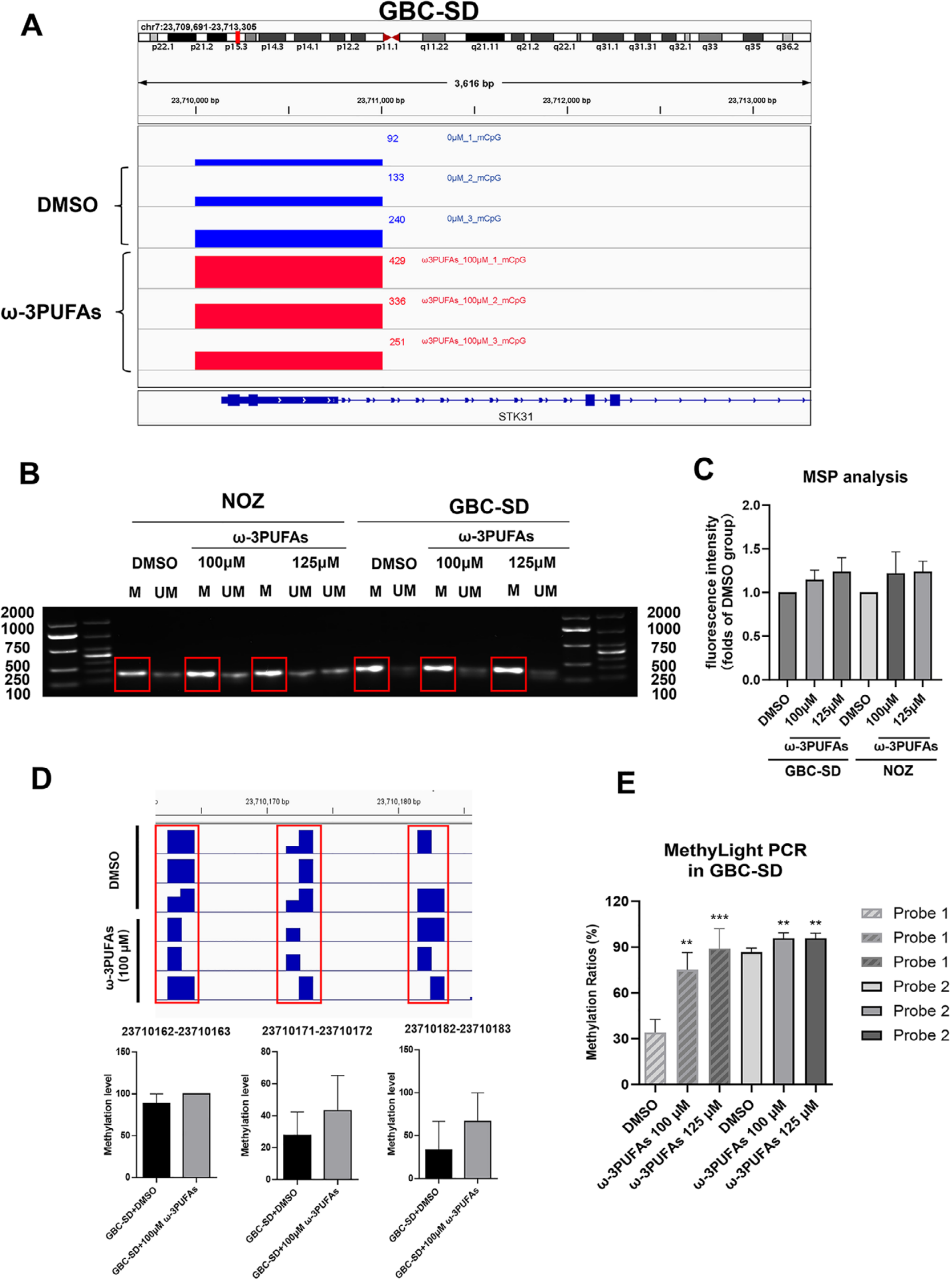
(A) Differential methylation of autosomal 7p15.3 locus in GBC‐SD cells after ω‐3PUFAs treatment. GBC‐SD cells incubated with 100 µM ω‐3PUFAs for 24 h, DNA was extracted and subjected to RRBS sequencing by the HiSeq4000 sequencing platform and 150PE machine strategy. The *STK31* promoter region of the 7p15.3 segment of the autosome was analyzed using IGV software, and it was found that the methylated reads at CpG sites (mCpG) of the samples treated with ω‐3PUFAs were significantly higher than those of the control group samples. (B and C) Methylation‐specific PCR (MSP) analysis of *STK31*. (B) Agarose gel electrophoresis of MSP products. M: product amplified with methylated‐specific primer; U: product amplified with unmethylated‐specific primer. GBC‐SD and NOZ cells were treated with 100 µM or 125 µM ω‐3PUFAs for 24 h, DNA was extracted and subjected to bisulfite conversion treatment. Subsequently, the samples were analyzed by MSP, and gel electrophoresis was performed to assess the presence of bands by UV transillumination. (C) The quantification of MSP results (*n* = 3). (D) Three CpG sites within the STK31 promoter exhibited significantly increased methylation levels after ω‐3PUFAs treatment, as revealed by RRBS.(E) Quantification of methylated *STK31* in GBC‐SD samples with or without ω‐3PUFAs by MethyLight PCR. Genomic DNA samples were bisulfite converted and analyzed by MethyLight PCR. Two probes were designed and employed in this assay. For each sample, the results were presented as the methylation ratios. All data were presented as mean ± SD from three independent biological replicates. Differences between treatment groups and control group were determined by one‐way ANOVA followed by post hoc Dunnett's test, **p < 0.01, ***p < 0.001.

Several studies have demonstrated that the expression of the *STK31* gene is controlled through DNA methylation [11, 12]. After treatment with ω‐3PUFAs, RRBS analysis revealed a significant increase in the methylated reads at CpG sites (mCpG) within the promoter region of *STK31* located on the autosomal 7p15.3 segment in GBC‐SD cells (Figure 3A). The MSP results also showed that after 24 h of treatment with 100 or 125 µM ω‐3PUFAs, the methylation level of the STK31 promoter region in NOZ and GBC‐SD cells exhibited an increasing trend (Figure 3B,C). RRBS analysis identified three CpG sites within the STK31 promoter region that exhibited significantly elevated methylation levels following ω‐3PUFAs treatment (Figure 3D). Two of these sites (23710171 and 23710182) were subsequently selected for the design of the TaqMan probe and primers (Table S1). Finally, we quantitatively detected the methylation level of *STK31* at these two sites in GBC‐SD cells using MethyLight PCR. The results showed that ω‐3PUFAs treatment significantly increased STK31 promoter methylation from 34.1% ± 20.91% to 89.1% ± 13.11% (Figure 3E, *p* < 0.01, 0.001).

**FIGURE 4:**
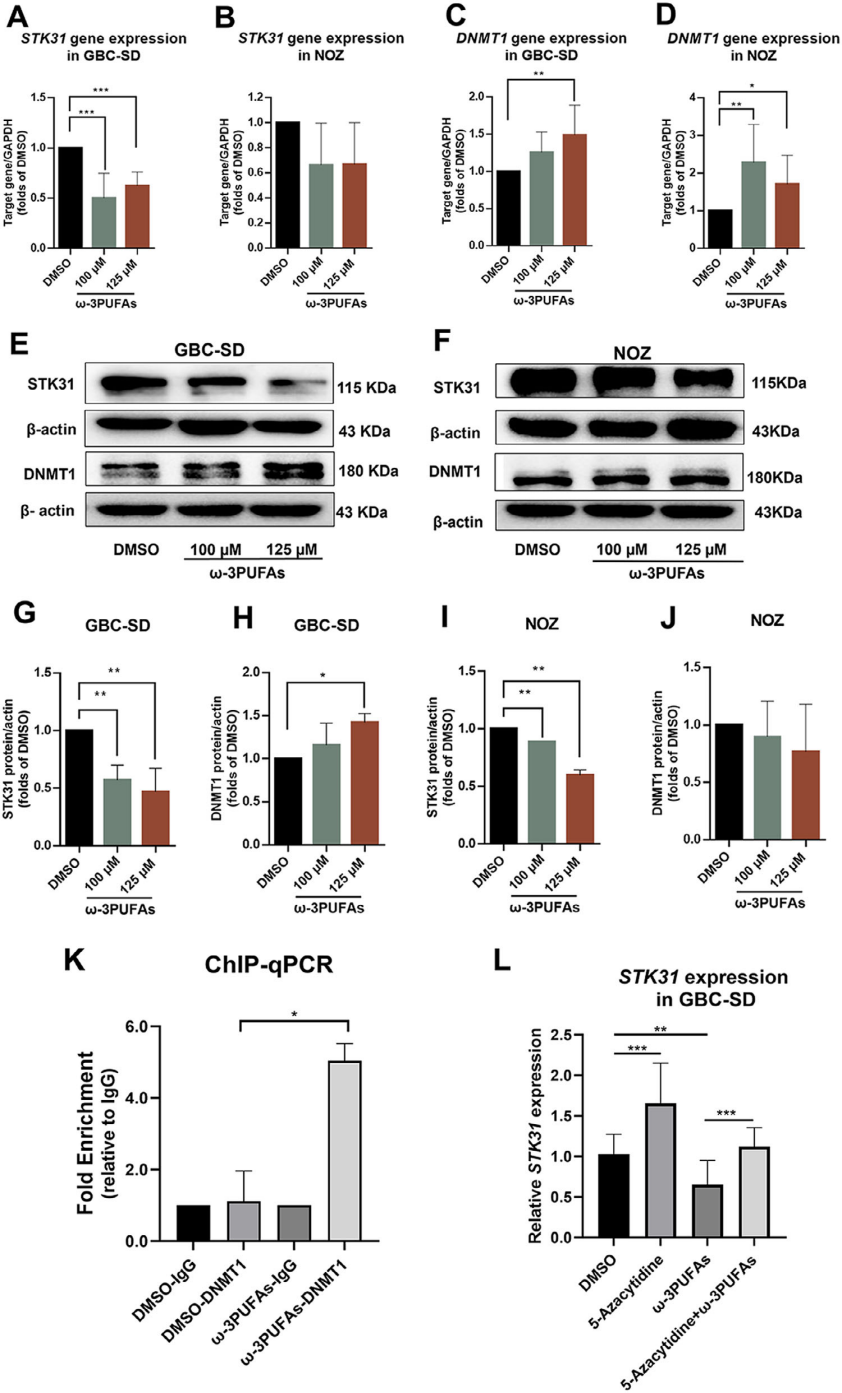
Effects of ω‐3PUFAs on STK31 and DNMT1 expression. (A–D) Quantitative real‐time PCR showed the expression of *STK31* and *DNMT1*. GBC‐SD and NOZ cells were treated with 100 µM or 125 µM ω‐3PUFAs for 24 h and then the cells were collected for RNA extraction. Data were normalized to corresponding *GAPDH* expressions as internal control. Data were from three independent biological replicates. (E and F) Western blot analysis of STK31 and DNMT1 expression. Representative immunoblots showed the effects of 100 or 125 µM ω‐3PUFAs on GBC‐SD and NOZ cellular expression of STK31and DNMT1. β‐actin expression was determined to confirm equal protein loading. (G–J) The histogram showed quantified results of protein levels, which were adjusted with corresponding β‐actin protein level and expressed as folds of control. Data were from three independent biological replicates. Differences between DMSO group and ω‐3PUFAs groups were determined by one‐way ANOVA followed by post hoc Dunnett's test, *p < 0.05, **p < 0.01, ***p < 0.001 as compared with vehicle control group. (K) GBC‐SD cells were treated with 100 µM ω‐3PUFAs for 24 h, ChIP‐qPCR was performed using anti‐DNMT1 antibody or IgG; primers spanning the *STK31* promoter region were used to detect the enrichment fold of DNMT1 at the *STK31* promoter region. Data were from two independent biological replicates. Differences among all groups were determined by one‐way ANOVA followed by post hoc Tukey's multiple comparison tests, *p < 0.05 as compared with DMSO vehicle control group. (L) GBC‐SD cells were treated with DMSO, 5 µM 5‐Aza, 100 µM ω‐3PUFAs, or a combination of 5‐Aza and ω‐3PUFAs for 24 h. Subsequently, the cells were harvested for RNA extraction and RT‐qPCR. Quantitative real time PCR analyses of mRNA of *STK31* gene were shown. Data were normalized to corresponding *GAPDH* expressions as internal control. Data were from three independent biological replicates. All data were presented as mean ± SD.***p < 0.001 as compared with ω‐3PUFAs group; **p < 0.01, ***p < 0.001 as compared with DMSO group by Student's *t*‐test.

DNMT1 is the key cellular methyltransferase, responsible for both de novo and maintenance methylation, and is essential for preserving gene methylation patterns in mammals [15]. Previous study also found that ω‐3PUFAs could increase the expression of DNMT1, thereby maintaining gene methylation and contributing to anti‐colorectal cancer effect [16]. Hence, during RT‐qPCR‐based detection of *STK31* expression, we further evaluated the effect of ω‐3PUFAs on *DNMT1* expression. Compared with the DMSO control group, 24 h ω‐3PUFA treatment markedly reduced *STK31* mRNA levels in GBC cells while concomitantly upregulating *DNMT1* expression significantly (Figure 4A–D, *p* < 0.05, 0.01). Additionally, western blot analysis showed that ω‐3PUFAs downregulated STK31 protein expression in both GBC‐SD and NOZ cells. Simultaneously it upregulated DNMT1 protein expression in GBC‐SD cells, whereas no significant change was observed in NOZ cells (Figure 4E–J). To explore the cell line‐specific discrepancy, we examined DNMT3B expression. As shown in Figure S1, DNMT3B expression was significantly upregulated by 100 µM ω‐3PUFAs in NOZ cells (Figure S1, *p* < 0.01). These data suggested that ω‐3PUFAs might induce STK31 promoter methylation via distinct DNMT family members in NOZ cells. Subsequently, to investigate whether DNMT1 binds to the *STK31* promoter region, we performed ChIP‐qPCR to detect the enrichment of DNMT1 at the *STK31* promoter in GBC‐SD cells treated with 100 µM ω‐3PUFAs for 24 h. The results showed that ω‐3PUFAs increased the enrichment levels of DNMT1 at the *STK31* promoter (Figure 4K). To further verify that the expression of *STK31* is indeed dependent on methylation regulation, we pretreated GBC‐SD cells with the methylation inhibitor 5‐Aza, followed by treatment with ω‐3PUFAs. The results showed that 5‐Aza alone significantly promoted the expression of *STK31*. Moreover, 5‐Aza partially reversed the inhibitory effect of ω‐3PUFAs on *STK31* expression (Figure 4L).

### 3.4 Proteomic Analysis of Downstream Mechanisms by Which ω‐3PUFAs Suppress GBC via STK31

**FIGURE 5:**
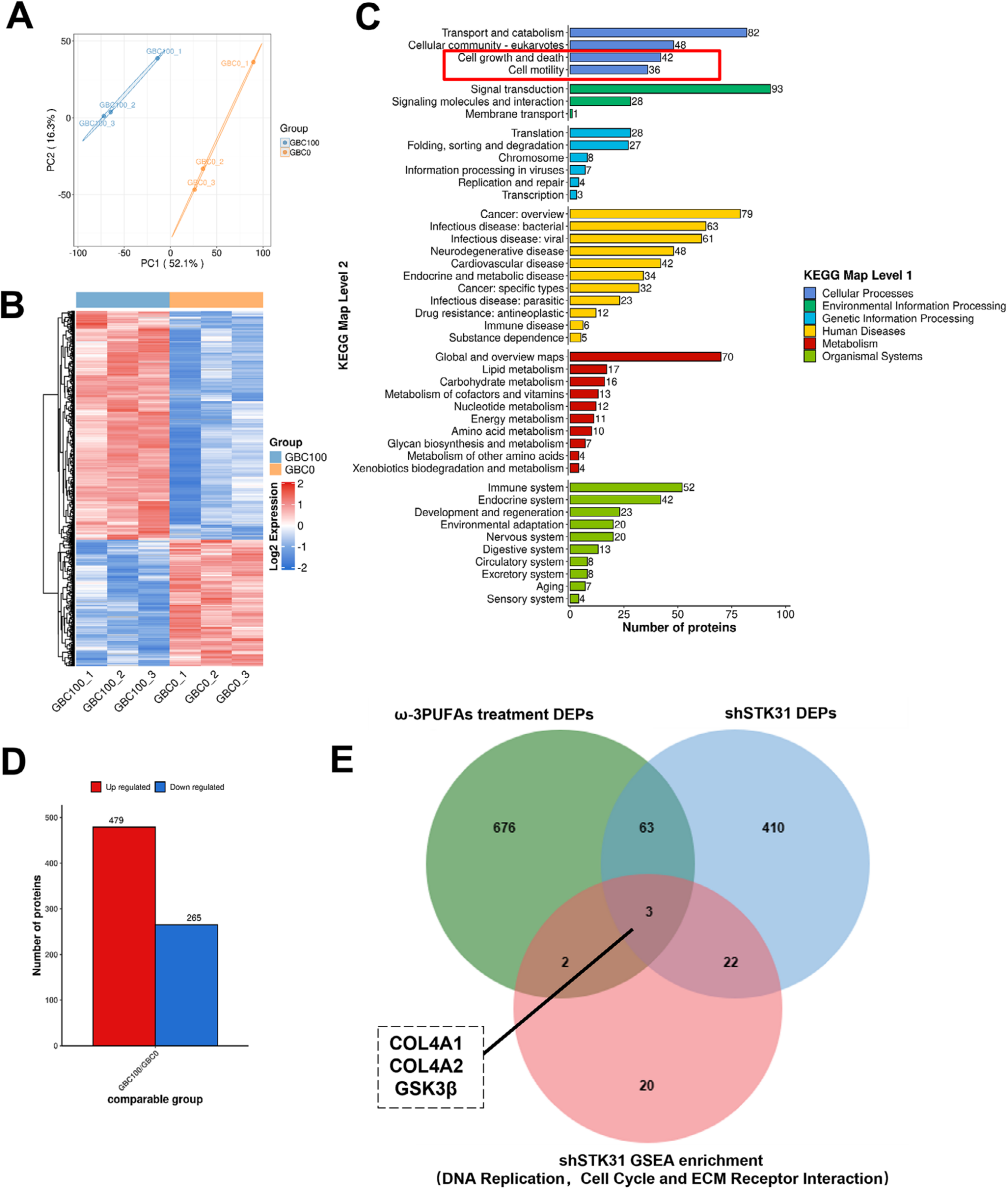
Proteomics analysis of GBC‐SD cells treated with ω‐3PUFAs. (A) Principal component analysis (PCA) of proteins identified by proteomics in GBC‐SD cells treated with or without ω‐3PUFAs. Each point represents an individual biological replicate, *n* = 3. Values in parentheses indicate the percentage of total variance explained by each principal component. (B) Heatmap showed the significantly up‐(red) and down‐(blue) proteins in GBC‐SD cells after treatment with 100 µM ω‐3PUFAs or vehicle (Ctr) for 24 h detected by proteomics; criteria: |FC| ≥ 1.5, *p *< 0.05. Only proteins with quantifiable values in at least two‐thirds of the total samples were included. Each row represents a differentially expressed protein, and each column represents an individual biological replicate. Red color indicates high expression, blue indicates low expression, and gray represents missing or non‐quantifiable values in the corresponding sample. (C) Kyoto Encyclopedia of Genes and Genomes (KEGG) pathway analysis of differentially enriched proteins in cells treated with ω‐3PUFAs versus vehicle control group. Data were from three independent biological replicates. The horizontal axis represented the number of DEPs within each category, while the vertical axis displayed the secondary functional classifications under the primary KEGG pathways. (D) Bar chart displayed the total number of significantly upregulated and downregulated proteins in cells after treatment with ω‐3PUFAs. Data were from three independent biological replicates. (E) Venn diagram comparing proteins that were significantly differential expressed (total 744 proteins) in ω‐3PUFAs treated GBC‐SD cells compared to vehicle control group (|FC| > 1.5; *p* *<* 0.05), differential expressed proteins in shSTK31 knock‐down GBC‐SD cells (total 498 proteins, |FC| > 1.5; *p* *<* 0.05), those with significantly enrichment score (total 47 proteins, *p* *<* 0.05) through gene set enrichment analysis (GSEA) using the fold change of protein inside the DNA Replication, cell cycle and ECM receptor interaction pathway in shSTK31 knock‐down GBC‐SD cells. Intersection of the above proteomics results for the potential down‐regulated proteins by ω‐3PUFAs through *STK31* with corresponding functions identified 3 proteins, including COL4A1, COL4A2, and GSK3β.

To further elucidate the downstream mechanisms through which ω‐3PUFAs inhibited GBC via *STK31*, proteomics and bioinformatics analysis were utilized to screen for potential molecules. Protein samples collected from GBC‐SD cells 24 h after ω‐3PUFAs treatment, along with samples obtained from the same cell line with *STK31* knockdown (from another study of ours), were both subjected to proteomic analysis. For the samples treated with ω‐3PUFAs, we performed proteomic analysis on three independent biological replicates and applied PCA for dimensionality reduction and visualization. As shown in Figure 5A, PC1, as the largest source of variation (52.1%), primarily represents the robust and consistent proteomic response induced by ω‐3PUFAs treatment. The ω‐3PUFAs‐treated samples exhibited excellent reproducibility, as evidenced by their tight clustering, and were clearly separated from the vehicle control group along the PC1 axis.

Additionally, a heatmap was generated to visualize the relative expression levels of the union of all differentially expressed proteins (DEPs) identified in the ω‐3PUFAs ‐treated groups versus the vehicle control groups (|FC| ≥ 1.5, *p *< 0.05). This visualization illustrated the clustering relationships of the relative expression levels of the DEPs across different samples. As shown in Figure 5B,D, bioinformatics analysis indicated that treatment with ω‐3PUFAs was associated with the upregulation of 479 proteins and the downregulation of 265 proteins. To further obtain relevant biological information, DEPs were also used for functional analysis with KEGG pathway. Our results revealed that a significant enrichment was observed for genes involved in pathways related to Cell growth and death, as well as Cell motility (Figure 5C). This finding was consistent with our previous biological functional assays of ω‐3PUFAs in GBC cells.

Subsequently, to identify the downstream molecules through which ω‐3PUFAs act via *STK31*, we performed a Venn diagram analysis on three protein sets: the 744 DEPs following ω‐3PUFAs treatment, the 498 significantly altered proteins after *STK31* knockdown, and the 47 proteins whose corresponding genes were significantly enriched in GBC proliferation and migration pathways (including DNA Replication, Cell Cycle and ECM Receptor Interaction) in gene set enrichment analysis (GSEA) after *STK31* knockdown. The intersection of the three datasets identified 3 overlapping proteins (GSK3β, COL4A1, and COL4A2), representing potential downstream targets of *STK31* regulated by ω‐3PUFAs (Figure 5E).

### 3.5 GSK3β And Its Downstream Molecules Involved in ω‐3PUFAs'effect on GBC Cells

**FIGURE 6:**
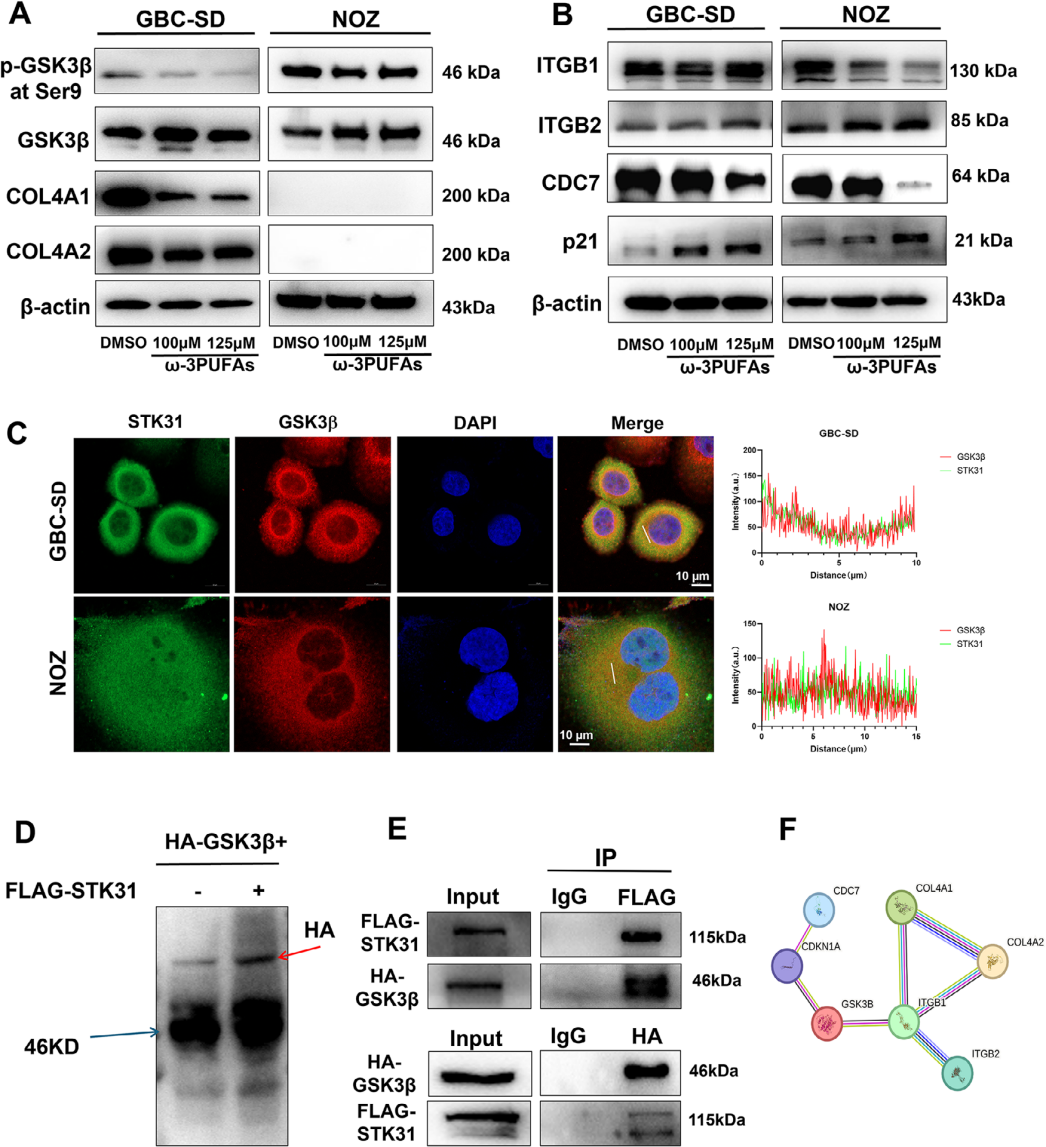
Western blot validation of molecules obtained from proteomics and bioinformatics analysis, and their associated protein expression related to proliferation and migration functions. (A and B) The representative immunoblots showed the effects of ω‐3PUFAs on the expression of GSK3β, p‐GSK3β, COL4A1, COL4A2, ITGB1, ITGB2, CDC7, and p21. β‐actin expression was assessed as an internal control for equal protein loading. (C) The expression and localization of STK31 and GSK3β in cells were detected by using immunofluorescence staining and confocal microscopy. Quantitative colocalization analysis performed using ZEISS ZEN 3.8 software. Scale bar = 10 µm. Representative images from three independent experiments. (D) Phos‐tag SDS‐PAGE and subsequent transfer analysis revealed that the addition of STK31 led to an increase in GSK3β phosphorylation. Representative images from three independent experiments. (E) The exogenous interaction between Flag‐STK31 and HA‐GSK3β in GBC‐SD cells was assessed by Co‐IP. Representative images from three independent experiments. (F) STRING database was applied for protein–protein interaction analysis, where the width of the connecting lines reflects the interaction strength.

Given that *STK31* is a serine/threonine kinase that regulates the expression of GSK3β, we further investigated whether GSK3β is a substrate of *STK31* and whether ω‐3PUFAs affect its phosphorylation through *STK31* expression. Western blot results confirmed that ω‐3PUFAs significantly increased the expression of GSK3β and decreased COL4A1 expression (Figures 6A and S2B,C), which were consistent with the proteomics data. Meanwhile, ω‐3PUFAs suppressed the phosphorylation of GSK3β (at Ser9) (Figures 6A and S2A). Interestingly, COL4A1 and COL4A2 were not expressed in NOZ cells (Figures 6A and S2C,D), indicating that ω‐3PUFAs did not regulate cell migration through this mechanism. Our previous study found that knocking down *STK31* significantly inhibited the expression of the integrin family, which played a pivotal role in cell migration [17]. Therefore, we further examined whether the expression of ITGB1 and ITGB2 in NOZ cells is affected by ω‐3PUFAs. The results showed that ω‐3PUFAs significantly downregulated ITGB1, but not ITGB2 in NOZ cells (Figures 6B and S2E,F). Study has found that inhibition of GSK3β phosphorylation can lead to an increase in p21 levels, thereby regulating cell cycle arrest [18]. CDC7 was a potential target gene which was regulated by p21 pathway [19] and our previous study also showed that knockdown of STK31 in GBC cells significantly inhibited CDC7 expression. Therefore, in this study, we also examined the regulatory effect of ω‐3PUFAs on CDC7 and p21. Western blot results demonstrated that it significantly suppressed CDC7 expression while concurrently increasing p21 expression (Figures 6B and S2G,H).

Furthermore, IF staining followed by confocal microscopy confirmed that STK31 and GSK3β were predominantly expressed in the cytoplasm and/or nucleus within the cells, demonstrating significant co‐localization (Figure 6C). The Phos‐tag SDS‐PAGE assay demonstrated that GSK3β phosphorylation occurred in the presence of STK31 (Figure 6D). Co‐IP assays demonstrated a direct interaction between STK31 and GSK3β (Figure 6E). The STRING database was utilized to depict the interaction relationships among the above proteins (Figure 6F). Based on these findings, we propose that ω‐3PUFAs inhibit STK31 expression, thereby altering GSK3β phosphorylation. Consequently, GSK3β‐mediated regulation of COL4A1/ITGB1 suppresses cell migration, while its effects on p21 and CDC7 contribute to cell cycle arrest and inhibition of cell proliferation.

### 3.6 ω‐3PUFAs Treatment Suppressed the Growth of GBC‐SD and NOZ Human Gallbladder Tumor Xenografts Through the Inhibition of STK31

**FIGURE 7:**
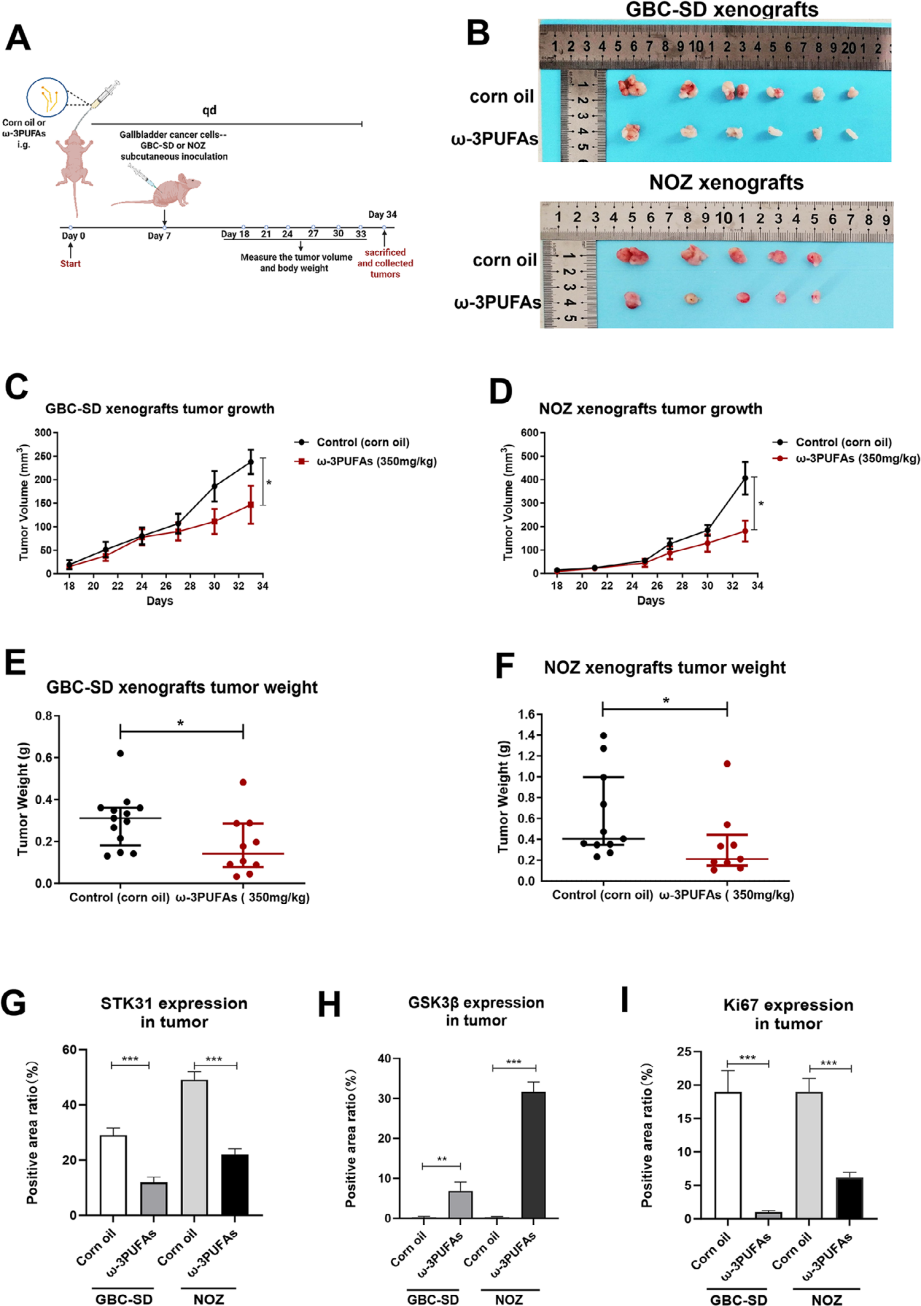
Effects of ω‐3PUFAs on GBC‐SD or NOZ human GBC xenograft models. (A) Experiment timeline of the establishment of the in vivo models and ω‐3PUFAs treatment in nude mice. ω‐3PUFAs (350 mg/kg) or vehicle control group (corn oil) pretreatment was oral administrated to mice each day. After 7 days, mice were injected subcutaneously with tumor cells. ω‐3PUFAs treatment was continued daily until the 33rd day, then the mice were sacrificed, and tumor samples were collected (Created in https://BioRender.com). (B) The representative images of the GBC tumors dissected from the mice. (C and D) Effects of ω‐3PUFAs on growth of xenograft tumors. Data were presented as mean ± SEM, GBC‐SD control group (*n* = 13), GBC‐SD ω‐3PUFAs group (*n* = 10); NOZ control group (*n* = 11), NOZ ω‐3PUFAs group (*n* = 9).Comparing with Student's *t*‐test, *p < 0.05. (E and F) The weight of dissected GBC‐SD and NOZ tumor was determined by nonparametric Mann–Whitney *U* test, *p < 0.05. Data were presented as median ± IQR (center values indicate median; error bars represent interquartile range, IQR) due to non‐normal distribution. GBC‐SD control group (*n* = 13), GBC‐SD ω‐3PUFAs group (*n* = 10); NOZ control group (*n* = 11), NOZ ω‐3PUFAs group (*n* = 9). (G–I) Statistical analysis and the representative photos of immunohistochemical staining for STK31, GSK3β, and Ki‐67 in tumor tissues. Data were presented as mean ± SEM, GBC‐SD control group (*n* = 13), GBC‐SD ω‐3PUFAs group (*n* = 10); NOZ control group (*n* = 14), NOZ ω‐3PUFAs group (*n* = 13). Differences between treatment group and vehicle control group were determined by Student's *t*‐test, **p < 0.01, ***p < 0.001.

Since in vitro experiments have demonstrated that ω‐3PUFAs can inhibit the proliferation and migration of GBC cells, we next utilized the nude mouse xenograft models to further validate the inhibitory effect of ω‐3PUFAs on tumor growth in vivo (Figure 7A). The results showed that in both GBC‐SD and NOZ xenograft models, the tumor growth rate was significantly slower in the group orally administered with ω‐3PUFAs compared to the vehicle control group receiving corn oil. At day 33, the tumor growth inhibition rates were 38.6% for GBC‐SD and 50.2% for NOZ (Figure 7B–D). Meanwhile, no significant difference in body weight was observed between the vehicle control (corn oil) group and the ω‐3PUFAs‐treated group throughout the entire experimental period, indicating a favorable safety profile of ω‐3PUFAs (Figure S3A,B). Additionally, the ω‐3PUFAs treatment group in NOZ xenograft model showed a median tumor weight of 0.212 g (IQR: 0.149–0.443 g), compared to 0.404 g (IQR: 0.348–0.996 g) in the vehicle control group. In the GBC‐SD xenograft model, the median tumor weights were 0.311 g (IQR: 0.182–0.361 g) and 0.142 g (IQR: 0.078–0.287 g) for the control and treatment groups, respectively. These data demonstrated that the final tumor weights in ω‐3PUFAs‐treated group were significantly lower than those in the corn oil control group (Figure 7E,F, *p* < 0.05).

Immunohistochemical analysis of tumor tissues revealed that ω‐3PUFAs treatment led to a significant reduction in STK31 expression, which was accompanied by increased GSK3β expression and decreased Ki‐67 levels (Figures 7G–I and S3C–E). This cascade suggests that suppression of STK31 by ω‐3PUFAs activates GSK3β, thereby restraining cancer cell proliferation and ultimately inhibiting tumor growth. These in vivo results align with our in vitro functional and mechanistic findings.

### 3.7 ω‐3PUFAs Treatment Suppressed the Metastasis of NOZ Human Gallbladder Tumor Xenografts

**FIGURE 8:**
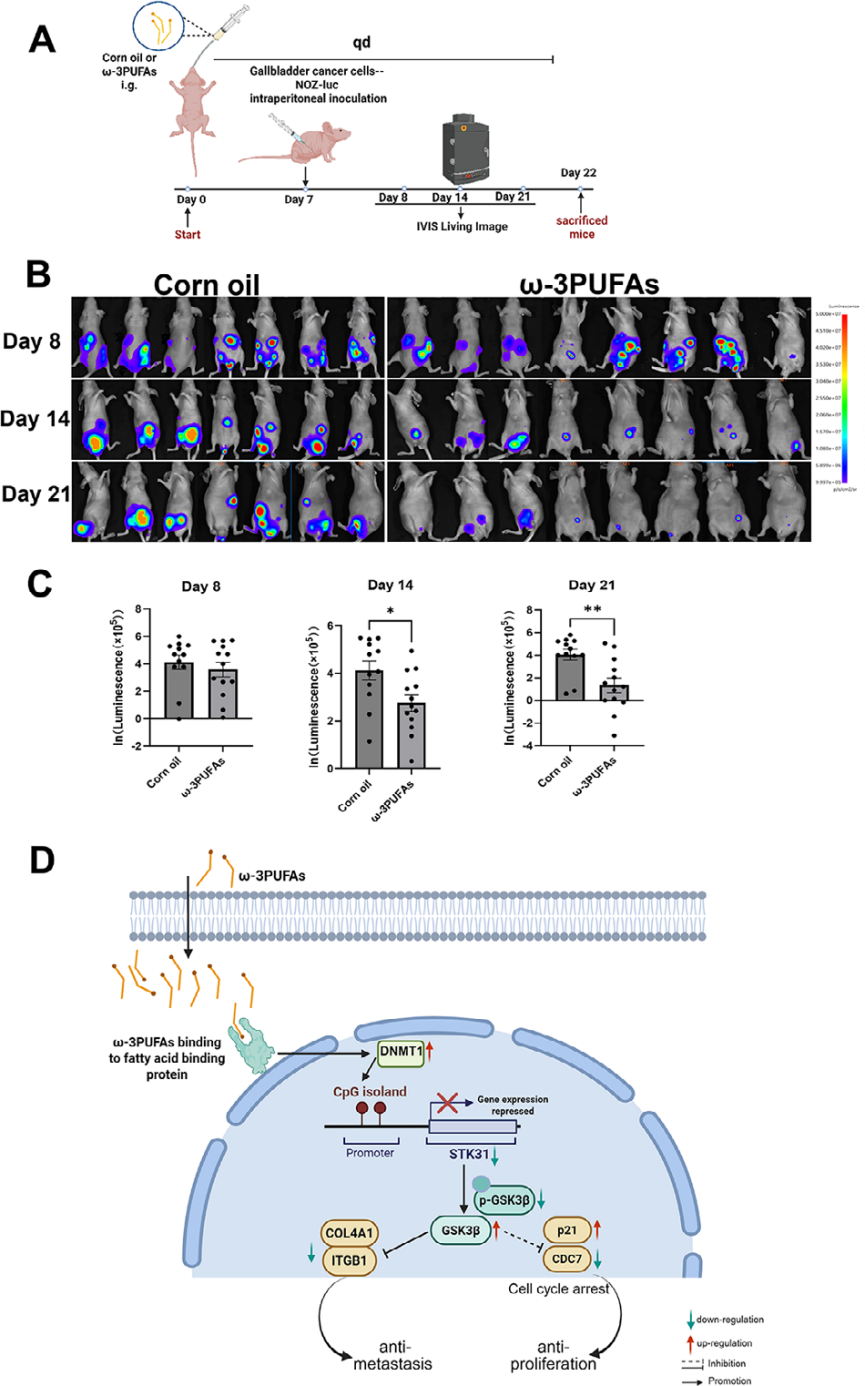
Effects of ω‐3PUFAs on intraperitoneal NOZ‐luc cells metastasis xenograft model. (A) Experiment timeline of the establishment of the in vivo metastasis model and IVIS detection (Created in https://BioRender.com). (B) The representative images from the mice monitored for the distribution of tumor cells with an IVIS Spectrum imaging system. (C) Quantitative analysis of tumor metastasis at different time points based on ROI measurements. Data were presented as mean ± SEM, *p < 0.05, **p < 0.01, as compared with control (corn oil group), unpaired Student's *t*‐test, corn oil group, *n* = 12; ω‐3PUFAs group, *n* = 13. (D) Schematic diagram of the potential mechanism by which ω‐3PUFAs inhibit GBC through promoting STK31 methylation to suppress its expression and regulating its downstream pathway (Created in https://BioRender.com).

To further evaluate the anti‐metastasis efficacy of ω‐3PUFAs in vivo, an intraperitoneal NOZ xenograft model (Figure 8A) was employed. As shown in Figure 8B,C, the NOZ tumor burden was quantified using IVIS imaging, which demonstrated no significant difference in the inoculation levels between the ω‐3PUFAs group and corn oil group on the second day after inoculation (Day 8 following ω‐3PUFAs administration). However, on Days 7 and 14 after tumor transplantation (Day 14 and Day 21 following ω‐3PUFAs administration), IVIS results demonstrated that ω‐3PUFAs significantly inhibited the metastasis of NOZ‐luc cells in vivo (Figure 8B,C, *p* < 0.05, 0.01).

## 4 Discussion

While a Mendelian randomization study indicated that ω‐3PUFAs may reduce the risk of cholecystitis, cholelithiasis, and disorders of the gallbladder, biliary tract; no robust statistical association was identified with GBC. This finding may, in part, reflect the methodological constraints of horizontal pleiotropy. Accordingly, further investigations are needed to elucidate the potential mechanisms linking polyunsaturated fatty acids to GBC risk [20]. Although our research result demonstrated that ω‐3PUFAs already exert significant anti‐proliferative effects at 25 and 50 µM in GBC‐SD cells, which falls within the physiologically achievable range (10–30 µM). However, the aforementioned concentrations of ω‐3PUFAs failed to effectively suppress cell proliferation in NOZ cells. Additionally, extensive other studies have shown that 100 µM DHA/EPA is a commonly used experimental concentration for mechanistic studies across multiple cancer types, including acute myeloid leukemia, multiple myeloma, pancreatic cancer, and prostate cancer [21, 22, 23, 24]. Therefore, we continued to use similar concentrations in our subsequent mechanism studies. Although the association between low‐dose supplementation of ω‐3PUFAs and disease risk remains to be further elucidated, the use of pharmacological doses of ω‐3PUFAs for disease treatment has been clearly established. Prescription ω‐3PUFAs formulations, such as Lovaza (EPA/DHA mixture) and Vascepa (pure EPA), have been approved by the U.S. FDA for the treatment of severe hypertriglyceridemia. Unlike dietary supplements, these pharmaceutical products are highly purified (> 90%) and supported by clinical evidence [4, 5]. Therefore, in this study, we employed an EPA/DHA mixture (1:1 ratio) to mimic the therapeutic application of ω‐3PUFAs. The dosage used in the nude mouse xenograft model (350 mg/kg) corresponds to a human equivalent dose within the clinically relevant range [25], thereby supporting the translational potential of our findings. Nevertheless, further pharmacokinetic investigations and clinical validation are required to confirm the applicability of these results to human therapy.

The overexpression of proto‐oncogenes is often associated with mechanisms such as gene amplification, transcriptional activation, or demethylation. For most proto‐oncogenes, especially those with promoters containing CpG islands, hypermethylation of the promoter region typically suppresses their expression, while hypomethylation promotes it [26, 27]. Therefore, certain GBC‐related proto‐oncogenes including *EGFR*, *RET*, *Myc*, *ERBB2*, *KRAS*, and so forth, were also evaluated after ω‐3PUFAs treatment based on RRBS results. The results indicated that exposure to ω‐3PUFAs also led to hypermethylation of the promoter regions of certain genes(Figure S4). Consequently, the methylation‐promoting function of ω‐3PUFAs appears to not be solely confined to the STK31; a further assessment of its regulatory impact on other genes is warranted in the future. The promoter region of the *STK31* gene contains at least one CpG island. Multiple studies have confirmed that when CpG island undergoes hypermethylation, the mRNA and protein expression levels of *STK31* are significantly reduced [11, 28]. DNA methylation is catalyzed by DNA methyltransferases (DNMTs), with DNMT1 functioning as the principal maintenance enzyme responsible for preserving CpG island methylation during DNA replication [15, 29, 30]. Therefore, in addition to assessing the methylation status of the *STK31*, we examined the impact of ω‐3PUFAs on DNMT1. ω‐3PUFAs markedly increased both DNMT1 mRNA and protein expression, with the most prominent effect observed in GBC‐SD cells. Consistently, ω‐3PUFAs also promoted DNMT1 enrichment at the *STK31* promoter region in this cell line. In contrast, these effects were less evident in NOZ cells, suggesting that the regulation of *STK31* methylation may involve distinct DNA methyltransferases across different GBC cell lines [26].

As a serine/threonine kinase, *STK31* influences downstream pathways through phosphorylation [31]. Proteomic analysis revealed that *STK31* affected GSK3β, and Co‐IP and other assays further demonstrated that this effect is direct. *STK31* inhibited the phosphorylation of GSK3β at Serine 9 (Ser9).

Our research also revealed, for the first time, that *STK31* exerted direct control over GSK3β by means of phosphorylation. GSK3β is a constitutively active kinase whose activity is inhibited by phosphorylation at Ser9 through multiple signaling pathways [32, 33]. Following treatment with ω‐3PUFAs, the decrease in phosphorylation of GSK3β (at Ser9) along with an increase in its total protein level indicated significant activation of the GSK3β pathway. Concurrently, the upregulation of p21 expression suggested that GSK3β may mediate cell cycle arrest by modulating p21, ultimately leading to the inhibition of cell proliferation—a finding consistent with previous studies [18, 34]. Furthermore, GSK3β can also reduce cell adhesion and migration by suppressing integrin family proteins [35, 36, 37] and integrins act as key receptors for the COL4A1/COL4A2 complex [38, 39], which provides a coherent explanation for our results. It is worth noting that, in addition to the differences in methylation mechanisms, the two GBC cell lines also exhibit distinct patterns in the molecules downstream of GSK3β that regulate cell metastasis and adhesion. This discrepancy may be attributed to the lack of COL4A1 and COL4A2 expressions in NOZ cells.

## 5 Conclusion

Taken together, these findings indicate that ω‐3PUFAs may be a potential therapeutic agent for GBC, as they can significantly promote the methylation of target gene such as *STK31* through DNMT1, thereby regulating the *STK31*/GSK3β axis to exert inhibitory effects (Figure 8D).

## Author Contributions

**Lin Li and Li‐Wei Liao**: writing – review and editing; **Shuang‐Xing Li and Wei‐Yu Zhu**: conceptualization, writing – original draft preparation; **Hai‐Ying Peng**: methodology; **Song‐Lin Yang and Bin Zhang**: investigation; **Ming‐Yao Meng**: project administration; **Zhu‐Ying Lin and Hui Gao**: data curation; **Yang‐Fan Guo**: visualization; **Yi‐Yi Zhao**: validation; **Yuan Liu**: resources; **Shan He**: formal analysis; **Xiao‐Dan Wang and Zong‐Liu Hou**: supervision; **Wen‐Ju Wang**: funding acquisition. All authors have read and agreed to the published version of the manuscript.

## Funding

This research was funded by National Natural Science Foundation of China (Grant numbers: 82260522 and 82460329), Key research and development plan of Yunnan Province (Grant number: 202403AC100002), and the Kunming Medical University Graduate Innovation Fund (Grant number: 2025S118).

## Ethics Statement

The animal study protocol was approved by the Animal Ethics and Welfare Committee (AEWC) of Yan'an Hospital Affiliated to Kunming Medical University (no. AEWC‐2022020) for studies involving animals.

## Consent

The authors have nothing to report.

## Conflicts of Interest

The authors declare no conflicts of interest.

## Supporting information




**Supporting File**: mnfr70522‐sup‐0001‐SuppMat.docx.

## Data Availability

The data that support the findings of this study are available on request from the corresponding author. The data are not publicly available due to privacy or ethical restrictions.
