# Supplementary material for: ω‐3PUFAs Inhibit Gallbladder Cancer Through Enhancing STK31 Methylation
Source: Mol Nutr Food Res. 2026 Jun 11;70(11):e70522. doi: 10.1002/mnfr.70522 (PMC13261096; doi:10.1002/mnfr.70522)
Supplement: Supplementary file 1 — Supporting File: mnfr70522‐sup‐0001‐SuppMat.docx. [file MNFR-70-e70522-s001.docx]

**Supplementary Figures**

**
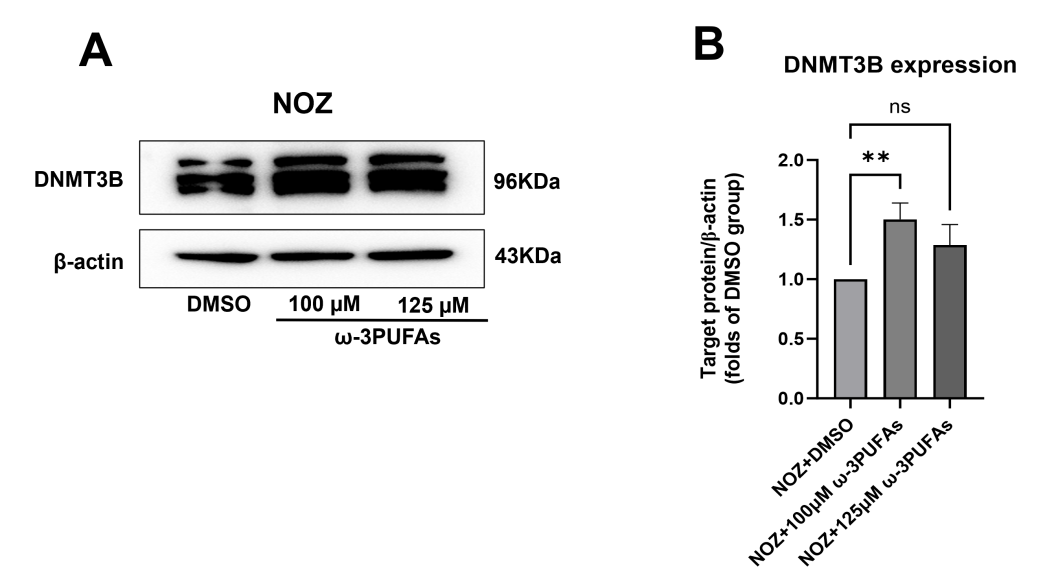
**

Supplementary Fig. 1 Effects of ω-3PUFAs on DNMT3B expression in NOZ cells. (A) Representative immunoblots showing the effects of 100 μM and 125 μM ω-3PUFAs on NOZ cellular expression of DNMT3B. β-actin expression was determined to confirm equal protein loading. (B) The histogram showed quantified results of protein levels, which were adjusted with corresponding β-actin protein level and expressed as folds of control. Data were presented as mean ± SD from three independent biological replicates. Differences between DMSO group and ω-3PUFAs groups were determined by one-way ANOVA followed by post-hoc Dunnett’s test, ***P*<0.01 as compared with vehicle control.

**
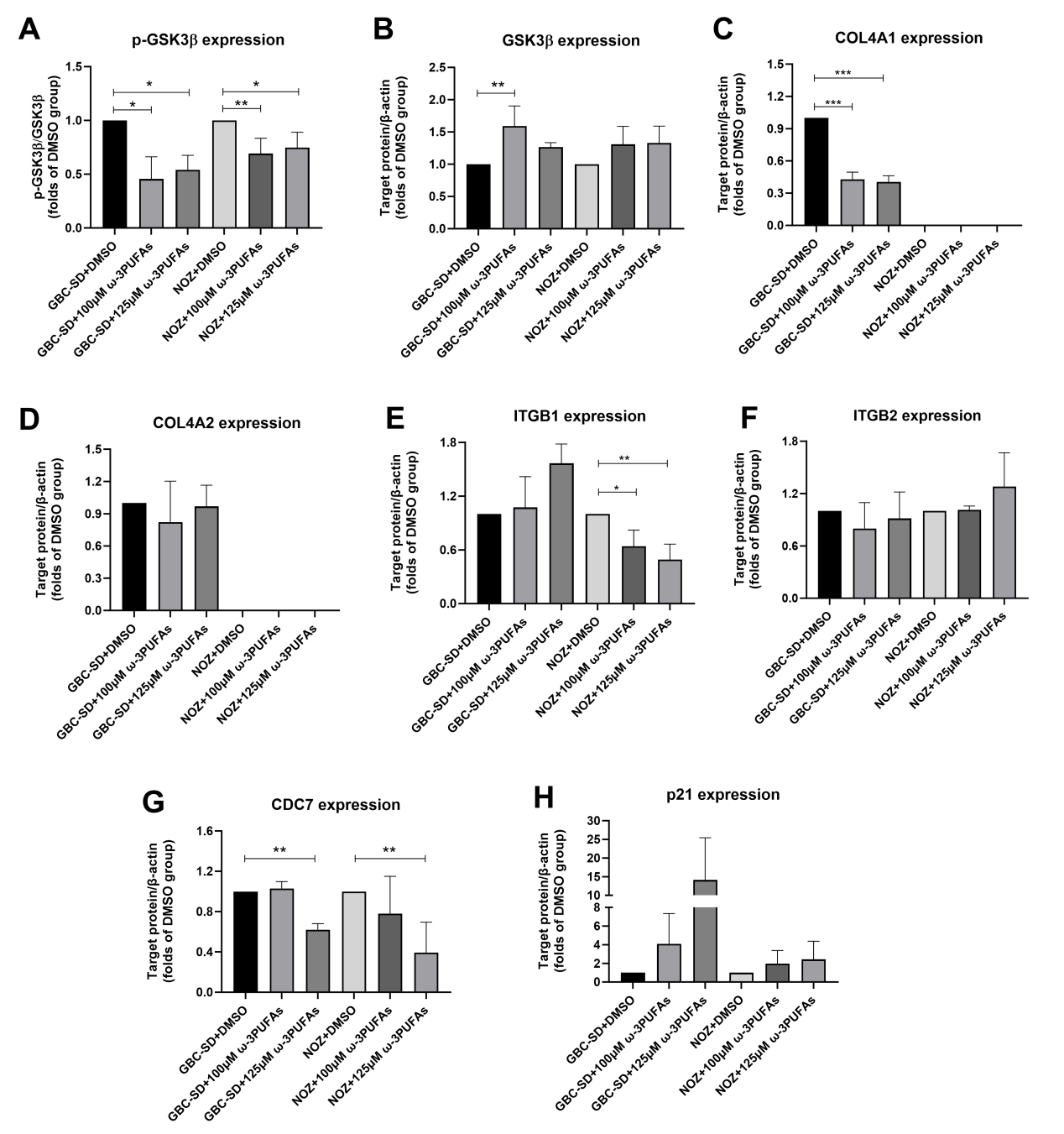
**

Supplementary Fig. 2 The histograms showed the quantified results of protein levels (p-GSK3β, GSK3β, COL4A1, COL4A2, ITGB1, ITGB2, CDC7 and p21), which were adjusted with corresponding β-actin protein level and expressed as fold of control. Data were presented as mean ± SD from three or four independent biological replicates. Differences between the treated and control groups were determined by one-way ANOVA followed by post-hoc Dunnett's test. **P*<0.05, ***P*<0.01, ****P*<0.001 vs vehicle control.


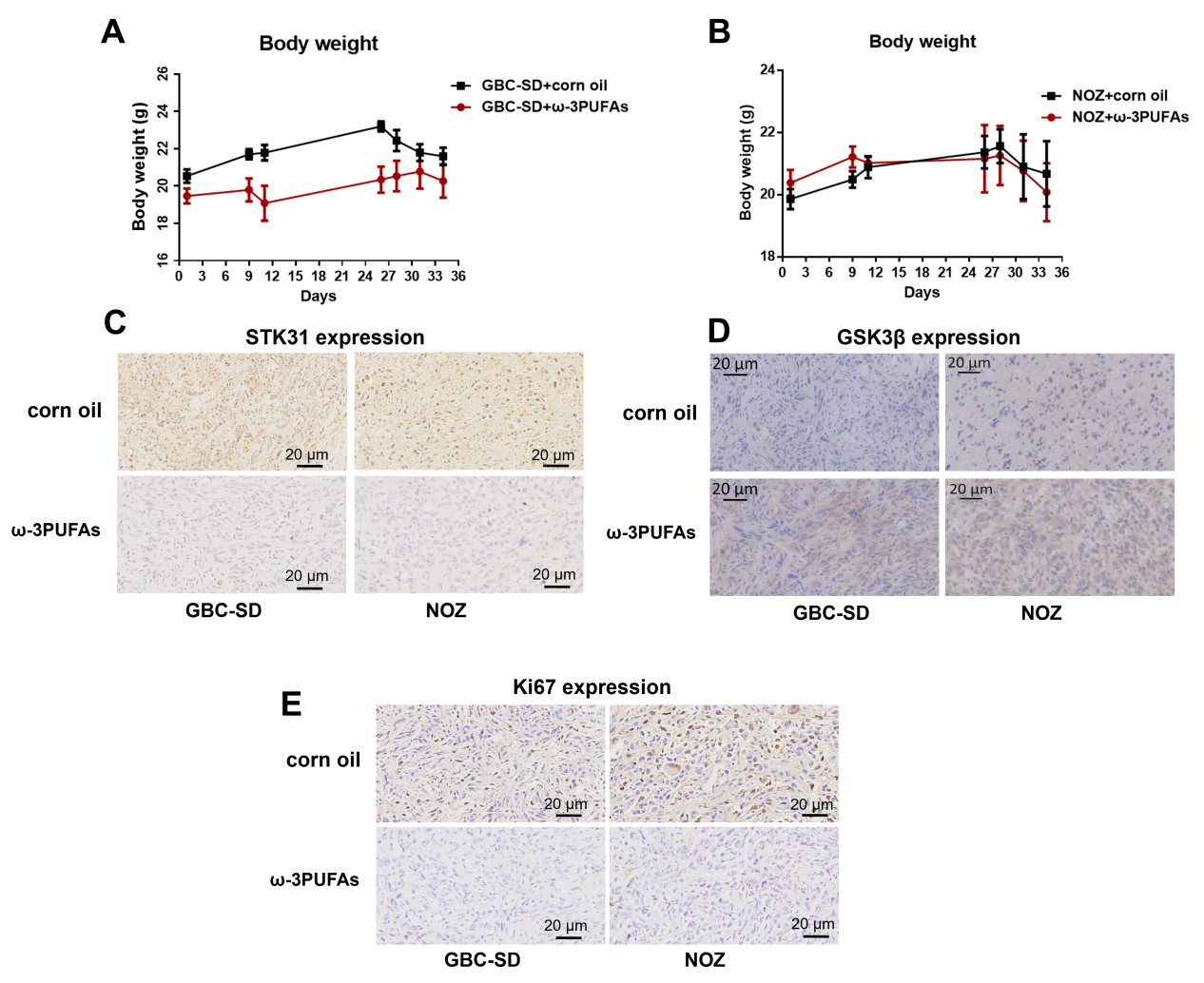


Supplementary Fig. 3 (A&B) The body weight of each mouse was monitored for 34 days in GBC-SD and NOZ xenograft animal models. Data were presented as mean ± SEM from biological replicates. GBC-SD control group(n=13), GBC-SD ω-3PUFA group(n=10); NOZ control group(n=11), NOZ ω-3PUFA group(n=9). (C-E) The representative photo of STK31, GSK3β and Ki-67 immunostaining.


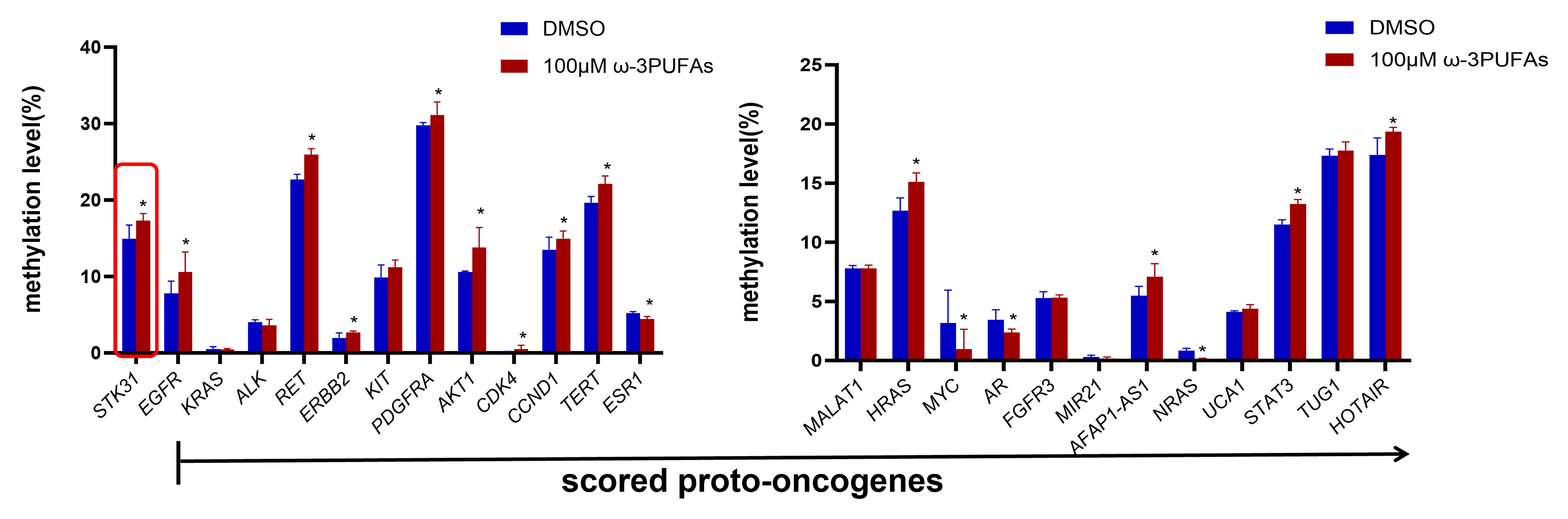


Supplementary Fig. 4 The methylation levels in the promoter regions of the top 30 GBC-related proto-oncogenes (identified from the database <https://www.genecards.org/>) were analyzed by RRBS following treatment with ω-3 PUFAs. Differential methylation analysis between the treated group and DMSO group were carried out using the R package MethylKit and determined by Fisher's exact test and Stouffer's meta-analysis method, **P*<0.001 vs DMSO group. Data were from three independent biological replicates.


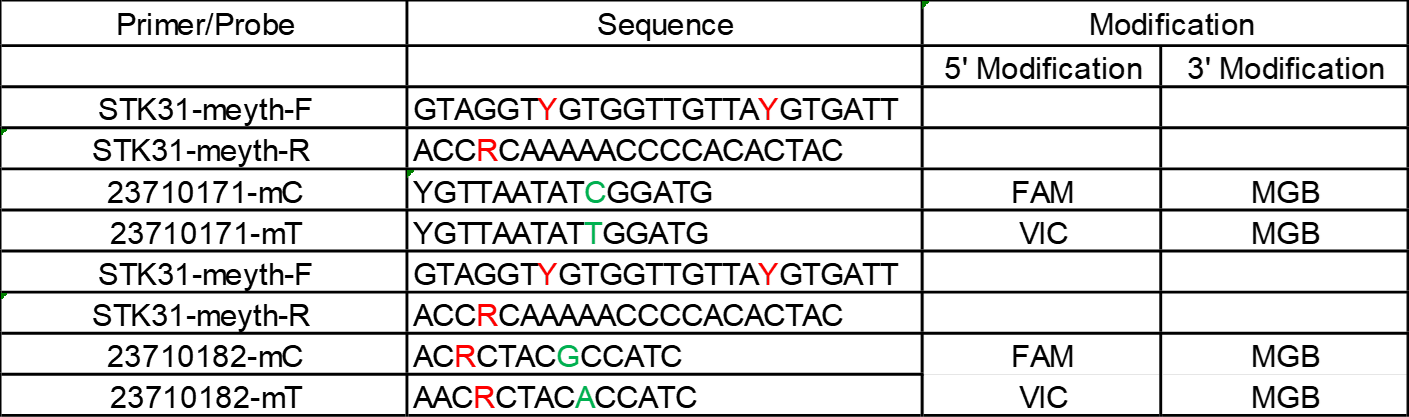


Supplementary Table 1 The primers/probes to detect the methylation levels of *STK31* promotor region by MethyLight PCR.
